# Risk factors for early revision after total hip and knee arthroplasty: National observational study from a surgeon and population perspective

**DOI:** 10.1371/journal.pone.0214855

**Published:** 2019-04-09

**Authors:** Alex Bottle, Sunny Parikh, Paul Aylin, Mark Loeffler

**Affiliations:** 1 Dr Foster Unit at Imperial College London, Department of Primary Care and Public Health, School of Public Health, London, United Kingdom; 2 Colchester General Hospital, Colchester, United Kingdom; Monash University, AUSTRALIA

## Abstract

**Aims:**

To identify predictors of early revision (within 3 years of the index operation) for hip and knee replacement (HR, KR) from both surgeon and population perspectives.

**Patients and methods:**

Hierarchical logistic regression on national administrative data for England for index procedures between April 2009 and March 2014.

**Results:**

There were 315,273 index HR procedures and 374,530 index KR procedures for analysis. Three-year revision rates were 2.1% for HR and 2.2% for KR. The highest odds ratios for HR were for 3+ previous emergency admissions, drug abuse, Parkinson’s disease, resurfacing and ages under 60; for KR these were patellofemoral or partial joint replacement, 3+ previous emergency admissions, paralysis and ages under 60. Smaller effects were found for other comorbidities such as obesity (HR) and diabetes (KR). From a population perspective, the only population attributable fractions over 5% were for male gender, uncemented total hip replacements and partial knee or patellofemoral replacements.

**Conclusions:**

Meeting the rising demand for revision surgery is a challenge for healthcare leaders and policymakers. Our findings suggest optimising patients pre-operatively and improving patient selection for primary arthroplasty may reduce the burden of early revision of arthroplasty. Our study gives useful information on the additional risks of various comorbidities and procedures, which enables a more informed consent process.

**Clinical relevance:**

Surgeons should make patients with certain risk factors such as age and procedure type aware of their higher revision risk as part of shared decision-making.

## Introduction

Around 7 million Americans were living with a hip or knee replacement in 2010, with growing prevalence and a shift to younger ages [1]. In the UK, over 83,000 primary hip replacements (HR) and 91,000 knee replacements (KR) were registered in the National Joint Registry for England, Wales, Northern Ireland and the Isle of Man (NJR) in 2014, and 8,900 hips and 5,800 knee replacements were revised [2]. Osteoarthritis has shown the largest growth among the non-communicable diseases in the Global Burden of Disease project, with total disability-adjusted life years (DALYs) rising by 35% and age-standardised DALY rates by 4% between 1990 and 2015 [3]. It is therefore expected that there will be a corresponding increase in the number of primary and revision surgeries [4–6].

Arthroplasty revision is most commonly performed for lysis, infection, fracture and, in hips, dislocation [7]. Late revision is foreseeable given the limited longevity of implants mainly due to mechanical wear and loosening, whereas early revision is typically linked to infection and instability [8]. The clinical burdens of a revision surgery are enormous as it is technically difficult with lower success rates than primary arthroplasty. Aseptic revision for total hip arthroplasty costs £11,897 on average, and £21,937 for a septic case [9]. To minimise the expected increasing burden of revision, particularly in cases of early failure, a comprehensive understanding of the relevant risk factors is crucial. Despite a number of studies, this remains limited.

Joint replacement revision rates vary with the type of prosthesis used and the bearing surface, for example metal on metal implants [10,11]. Patient comorbidities and demographics also have an impact on the revision rate. A 2008 systematic review that considered studies on patient age, sex, race, body weight, socioeconomic status and work status found that younger age and male sex are associated with increased revision risk after total joint arthroplasty for hip or knee osteoarthritis [12]. However, this review did not consider comorbidities. Dy et al used New York and California statewide data, finding that younger age, Medicaid insurance and low hospital volume increased the ten-year risk of revision total hip arthroplasty [13]. Patients with hypothyroidism, depression, diabetes, COPD, obesity and fluid/electrolyte disorders were also at higher risk of revisions. The same group similarly examined the data for knee arthroplasty and found slightly raised odds for revision within 10 years for COPD, diabetes, coagulopathy and depression [14]. They did not look at procedure subtype or dementia.

It is important to distinguish “early” from later revisions. Patient and surgeon expectations are high given the durability of conventional metal-on-plastic designs [15,16]. Early revisions often result in devastating outcomes, but patient factors associated with an increased risk of early revision are poorly understood, particularly in elderly patients [17]. Bozic et al analysed the 5% sample of the Centres for Medicare & Medicaid Services (CMS) Medicare claims database (excluding patients aged under 65) and found that depression, rheumatological disease, psychoses, renal disease, chronic urinary tract infection, and congestive heart failure were associated with one-year revision hip replacement [17]. Melvin et al took a 5-year period to mean “early” [18]. The main reasons for revision had changed since their earlier study covering 1986–2000, as instability had become much less common. Periprosthetic fracture and metallosis (failures related to the metal on metal articulation) had become much more common, and aseptic loosening and infection accounted for similar proportions of early revisions as in their earlier study. Reviewing the Norwegian Arthroplasty Register from 1987 to 2007, Fevang et al reported an overall decrease in revision rates but a shift towards a higher percentage of revisions being performed early, mainly due to dislocation and infection [19]. These studies point to a need to continue to monitor the predictors of early revisions and that evidence from other countries should be gathered.

To our knowledge, little work on early revision predictors has come from the UK. We used national administrative data to determine the relationship between early revision of both hip and knee arthroplasty within 3 years of the primary procedure and patient demographics, comorbidities and procedure subtype for patients of all ages.

The standard way of reporting regression results for risk factors is odds ratios or hazard ratios. These are important from both the patient and surgeon perspective and we report them here. However, the population perspective that takes risk factor prevalence into account is frequently missing from such studies. We have therefore also reported population attributable fractions (PAF) for each factor in our study.

## Methods

### Data

Hospital Episodes Statistics (HES) is the national hospital administrative database for England, covering all NHS (public) hospitals. Each row of the database covers a “finished consultant episode”, the period during which a patient is under the care of a senior physician (consultant). Using HES’s own patient identifier “HESID”, which involves a hierarchy of their NHS Number (unique to each person) where given and their date of birth, sex and postcode if not given, we linked records into admissions and linked admissions ending in transfer to another hospital into “superspells”; we analysed superspells and will refer to them as simply “admissions”. Outcome data were assumed to be complete for every patient (or at least assumed to be missing completely at random). With a national database, the main losses to follow-up occurs when patients move or are treated abroad. HES has 20 diagnosis fields, which use ICD10 coding, and 24 procedure fields, which uses the UK’s own “OPCS” coding system. The diagnosis fields capture comorbidities and complications. Each admission and procedure is dated. Urgency is indicated by the method of admission field (elective or emergency). We extracted records with discharge dates in the financial years 2006/7 to 2013/14 inclusive. Further information is given on NHS Digital’s website (https://digital.nhs.uk/).

### Definition of index and revision procedures

Index elective (planned) hip and knee replacements (HR, KR) for any indication were identified using the method of admission field and any of the OPCS codes given in the Appendix in any procedure field. Index procedures were those with any of these codes between April 2009 and March 2014 in order to allow three full years of follow-up for all patients. Revisions were defined as any of the list of OPCS codes in the NJR’s definition in their report (S1 Table). The few missing procedure dates were taken to be the date of admission. Joint laterality was recorded in 99% of index procedures; where this was unknown, we assumed that the revision related to the most recent joint replacement. Revisions were included at any time between one day and three years after the index procedure. Revisions were matched on laterality to the index.

### Statistical analysis

Our previous work comparing two popular comorbidity indices, Charlson [20] and Elixhauser [21], recommended using the Elixhauser set plus dementia [22,23]. We therefore first tabulated each of this combined set of comorbidities and excluded those with a prevalence of <0.1%. We then built three-level multiple logistic regression models using the remaining variables plus age group, sex, Carstairs socio-economic deprivation fifth, number of admissions in the year before the index operation, with patient, surgeon and hospital as the three levels [24]. To aid model fitting, consultant codes with five or fewer procedures between 2009/10 and 2013/14 were combined into one new pseudoconsultant. Likewise, we combined hospital trusts fewer than 50 procedures during that time into one new pseudohospital. Model fit was checked using global goodness-of-fit tests and by inspecting residuals and standard errors. The effect of mortality was checked by running cause-specific survival analysis with death as the competing event for revision and found to be limited.

The population attributable fraction was calculated for each covariate with a significantly (p<0.05) elevated odds ratio using the formula by Levine [25]; adjusted odds ratios stood in for relative risks, for which they will be a good approximation given that the outcome here is rare.

## Results

There were 325,377 index HR procedures and 387,222 index KR procedures during the five years 2009/10 to 2013/14. 10,056 HRs and 12,422 KRs were excluded due to invalid or unknown consultant team codes. 62 HRs and 270 KRs of unknown subtypes were also dropped. This left 315,273 HRs among 2,287 surgeons and 374,530 KRs among 2,179 surgeons for analysis. Less than 0.1% of index procedures lacked an operation date (the admission date was used instead), and 1% of revisions had missing laterality.

There were 5,679 revisions within three years for a rate of 2.1% for HR and 7,792 revisions within three years for a rate of 2.2% for KR. Table 1 gives the patient characteristics. For hips, 87% of index admissions had a primary diagnosis of coxarthrosis, with polyarthrosis and pain in joint accounting for most of the remainder. For knees, 89% were for gonarthrosis, with most of the rest also accounted for by polyarthrosis and pain in joint; 0.8% were for rheumatoid arthritis. 74% of HR revisions had a primary diagnosis of prosthesis complications (ICD10 T80-88), with 43% mechanical complications and 20% infections; in addition, 10.5% were fractures, with the rest largely recorded as coxarthrosis. 82% of KR revisions had a primary diagnosis of prosthesis complications, with 34.6% mechanical complications and 24.2% infections; in addition, 1.1% were fractures, with the rest largely recorded as gonarthrosis.

**Table 1:** Patient characteristics.

Factor		N (%) for hips	N (%) for knees
Age	0–39	6138 (1.9)	1181 (0.3)
	40–44	5623 (1.8)	2616 (0.7)
	45–49	10033 (3.2)	7500 (2.0)
	50–54	17853 (5.7)	17386 (4.6)
	55–59	27787 (8.8)	31804 (8.5)
	60–64	45674 (14.5)	56439 (15.1)
	65–69	60580 (19.2)	70978 (19)
	70–74	68931 (21.9)	72123 (19.3)
	75–79	72429 (23.0)	62987 (16.8)
	80–84	65225 (20.7)	36613 (9.8)
	85–89	48470 (15.4)	13051 (3.5)
	90+	29608 (9.4)	1852 (0.5)
Sex	Male	188321 (59.7)	214173 (57.2)
	Female	126952 (40.3)	160357 (42.8)
Op subtype (hips)	Resurfacing	5803 (1.8)	
	Hybrid	44031 (14.0)	
	No cement	137989 (43.8)	
	Cement	125776 (39.9)	
	Partial, cemented	945 (0.3)	
	Partial, not cemented	729 (0.2)	
Op subtype (knees)	PFR		8274 (2.2)
	Partial (UKR)		23986 (6.4)
	Total		342270 (91.4)
Number of prior emergency admissions	0	286145 (90.8)	342486 (91.4)
	1	22998 (7.3)	26034 (7.0)
	2	4258 (1.4)	4337 (1.2)
	3+	1872 (0.6)	1673 (0.4)
Socio-economic deprivation: least deprived	1	75468 (23.9)	78848 (21.1)
	2	81846 (26.0)	90881 (24.3)
	3	69694 (22.1)	83425 (22.3)
	4	52543 (16.7)	68395 (18.3)
Most deprived	5	34959 (11.1)	52233 (13.9)
Unknown	6	763 (0.2)	748 (0.2)
Year	2009	58709 (18.6)	70617 (18.9)
	2010	61959 (19.7)	73617 (19.7)
	2011	63863 (20.3)	76746 (20.5)
	2012	63545 (20.2)	75604 (20.2)
	2013	67197 (21.3)	77946 (20.8)
Diabetes		29098 (9.2)	48297 (12.9)
Hypertension		138327 (43.9)	188914 (50.4)
Arrhythmias		23748 (7.5)	27004 (7.2)
Valvular disorders		6098 (1.9)	6374 (1.7)
Heart Failure		3450 (1.1)	3455 (0.9)
Peripheral vascular disease		3212 (1.0)	3532 (0.9)
Chronic lung disease		40039 (12.7)	51808 (13.8)
Lung circulation disorders		841 (0.3)	1587 (0.4)
Cancer without metastasis		3456 (1.1)	3098 (0.8)
Cancer with metastasis		642 (0.2)	249 (0.1)
Renal disease		10700 (3.4)	12453 (3.3)
Dementia		987 (0.3)	751 (0.2)
Psychoses		459 (0.1)	385 (0.1)
Alcohol abuse		6446 (2.0)	6536 (1.7)
Drug abuse		334 (0.1)	171 (<0.1)
Depression		8764 (2.8)	11064 (3.0)
Liver disease		1227 (0.4)	1251 (0.3)
Peptic ulcer		367 (0.1)	550 (0.1)
Paraplegia		538 (0.2)	640 (0.2)
Blood loss anaemia		165 (0.1)	104 (<0.1)
Deficiency anaemia		2486 (0.8)	3030 (0.8)
Fluid and electrolyte disorders		3479 (1.1)	3935 (1.1)
Hypothyroidism		20190 (6.4)	26477 (7.1)
Obesity		21797 (6.9)	37254 (9.9)
Other neuro diseases except Parkinson’s disease		3804 (1.2)	4572 (1.2)
Parkinson’s disease		1253 (0.4)	2268 (0.6)
Rheumatic disorders		11584 (3.7)	14584 (3.9)
Index admission primary diagnosis	Arthrosis	273371 (86.7)	337695 (90.2)
	Polyarthrosis	16819 (5.3)	15899 (4.3)
	Rheumatoid arthritis	1614 (0.5)	3327 (0.9)
	Trauma	658 (0.2)	n/a
	Other	22811 (7.2)	17609 (4.7)

PFR = patellofemoral replacement; UKR = unicondylar knee replacement

Tables 2 and 3 give the odds ratios. The significant variables for HR and KR are described below, firstly from a surgeon perspective with odds ratios and secondly from a population perspective with PAFs. Table 4 gives the PAFs for the statistically significant predictors.

**Table 2:** Odds ratios for 3-year revision rates for index HR.

Factor		OR for 3yr revision	P value
Age	0–39	1.20 (1.01 to 1.42)	0.043
	40–44	1.29 (1.08 to 1.54)	0.005
	45–49	1.27 (1.10 to 1.47)	0.001
	50–54	1.05 (0.93 to 1.19)	0.444
	55–59	1.05 (0.94 to 1.18)	0.366
	60–64	1	
	65–69	0.99 (0.89 to 1.08)	0.756
	70–74	0.93 (0.84 to 1.03)	0.159
	75–79	0.93 (0.84 to 1.03)	0.172
	80–84	0.92 (0.82 to 1.04)	0.178
	85–89	0.92 (0.78 to 1.07)	0.279
	90+	0.90 (0.66 to 1.23)	0.496
Sex	Male	1.19 (1.13 to 1.26)	<0.0001
	Female	1	
Op subtype	Resurfacing	1.90 (1.60 to 2.24)	<0.0001
	Hybrid	1.11 (1.00 to 1.22)	0.043
	No cement	1.37 (1.27 to 1.47)	<0.0001
	Partial HR, cemented	1.08 (0.70 to 1.65)	0.738
	Partial HR, not cemented	1.36 (0.87 to 2.13)	0.184
	Cement	1	
Number of prior emergency admissions	0	1	
	1	1.30 (1.18 to 1.42)	<0.0001
	2	1.35 (1.12 to 1.64)	0.002
	3+	1.85 (1.46 to 2.35)	<0.0001
Year	2009	1	
	2010	0.93 (0.85 to 1.00)	0.064
	2011	0.87 (0.80 to 0.94)	0.001
	2012	0.78 (0.72 to 0.85)	<0.0001
	2013	0.76 (0.70 to 0.83)	<0.0001
Dementia		1.63 (1.13 to 2.35)	0.010
Arrhythmias		1.13 (1.02 to 1.25)	0.016
Rheumatic disorders		1.15 (1.01 to 1.31)	0.041
Chronic lung disease		1.10 (1.02 to 1.19)	0.017
Lung circulation disorders		1.61 (1.08 to 2.39)	0.019
Other neuro diseases except Parkinson’s disease		1.79 (1.50 to 2.13)	<0.0001
Obesity		1.37 (1.25 to 1.51)	<0.0001
Fluid and electrolyte disorders		1.34 (1.07 to 1.67)	0.010
Alcohol abuse		1.31 (1.11 to 1.55)	0.001
Drug abuse		2.10 (1.33 to 3.31)	0.002
Depression		1.34 (1.17 to 1.54)	<0.0001
Parkinson’s disease		1.75 (1.26 to 2.41)	0.001
Index primary diagnosis:	Coxarthrosis	1	
	Polyarthrosis	1.15 (1.02 to 1.30)	0.018
	Rheumatoid arthritis	1.44 (1.04 to 1.98)	0.028
	Trauma	1.77 (1.13 to 2.77)	0.013
	Other	1.66 (1.52 to 1.81)	<0.0001

Variables with p>0.05 have been omitted from the table

**Table 3:** Odds ratios for 3-year revision rates for index KR.

Factor		OR for 3yr revision	P value
Age	0–39	1.05 (0.80 to 1.38)	0.737
	40–44	1.63 (1.37 to 1.95)	<0.0001
	45–49	1.47 (1.29 to 1.66)	<0.0001
	50–54	1.37 (1.24 to 1.50)	<0.0001
	55–59	1.25 (1.15 to 1.36)	<0.0001
	60–64	1	
	65–69	0.85 (0.78 to 0.91)	<0.0001
	70–74	0.75 (0.70 to 0.82)	<0.0001
	75–79	0.62 (0.57 to 0.68)	<0.0001
	80–84	0.51 (0.46 to 0.57)	<0.0001
	85–89	0.50 (0.42 to 0.60)	<0.0001
	90+	0.40 (0.25 to 0.65)	0.0002
Sex	Male	1.24 (1.18 to 1.29)	<0.0001
	Female	1	
Op subtype	PFR	5.16 (4.74 to 5.60)	<0.0001
	Partial	2.81 (2.61 to 3.02)	<0.0001
	Total	1	
Number of prior emergency admissions	0	1	
	1	1.34 (1.24 to 1.45)	<0.0001
	2	1.54 (1.29 to 1.83)	<0.0001
	3+	1.85 (1.44 to 2.38)	<0.0001
Socio-economic deprivation: least deprived	1	1	
	2	1.11 (1.03 to 1.19)	0.004
	3	1.11 (1.03 to 1.20)	0.005
	4	1.17 (1.08 to 1.26)	<0.0001
Most deprived	5	1.23 (1.13 to 1.33)	<0.0001
Unknown	6	0.70 (0.39 to 1.25)	0.223
Year	2009	1	
	2010	0.97 (0.91 to 1.05)	0.476
	2011	0.99 (0.92 to 1.06)	0.704
	2012	0.94 (0.88 to 1.02)	0.125
	2013	0.92 (0.85 to 0.99)	0.022
Arrhythmias		1.11 (1.01 to 1.22)	0.032
Chronic lung disease		1.13 (1.06 to 1.20)	0.0002
Paralysis		1.59 (1.04 to 2.42)	0.032
Renal disease		0.82 (0.70 to 0.95)	0.011
Fluid and electrolyte disorders		1.29 (1.03 to 1.61)	0.026
Parkinson’s disease		1.49 (1.15 to 1.94)	0.003
Diabetes		1.10 (1.03 to 1.18)	0.005
Index primary diagnosis:	Gonarthrosis	1	
	Polyarthrosis	1.04 (0.92 to 1.18)	0.491
	Rheumatoid arthritis	1.00 (0.77 to 1.30)	0.995
	Other	2.59 (2.40 to 2.79)	<0.0001

Variables with p>0.05 have been omitted from the table

**Table 4:** Prevalences and population attributable fraction estimates for p<0.05 predictors of early revisions after hip and knee replacements.

Factor	Prevalence in % (hips)	Prevalence in % (knees)	PAR (hips) as %	PAR (knees) as %
Age 0–39	1.9	0.3	0.4	-
Age 40–44	1.8	0.7	0.5	0.3
Age 45–49	3.2	2.0	0.9	0.6
Age 50–54	5.7	4.6	0.3	1.1
Age 55–59	8.8	8.5	0.5	1.2
Resurfacing	1.8	n/a	1.6	n/a
Hybrid	14.0	n/a	1.5	n/a
Not cemented	43.8	n/a	13.8	n/a
Partial, cemented	0.3	n/a	<0.1	n/a
Partial, not cemented	0.2	n/a	0.1	n/a
Cemented	39.9	n/a	n/a (reference)	n/a
PFR	n/a	2.2	n/a	7.6
Partial (UKR)	n/a	6.4	n/a	9.4
Total knee replacement	n/a	91.4	n/a	n/a (reference)
Females	59.7	57.2	n/a (reference)	n/a (reference)
Males	40.3	42.8	7.2	7.1
Quintile 4	-	18.3	-	1.5
Quintile 5 (most deprived)	-	13.9	-	1.8
No prior emergency adms	90.8	91.4	n/a (reference)	n/a (reference)
1 prior emergency adms	7.3	7.0	2.1	1.6
2 prior emergency adms	1.4	1.2	0.5	0.4
3+ prior emergency adms	0.6	0.4	0.5	0.2
Arrhythmias	7.5	7.2	0.9	0.1
Chronic lung disease	12.7	13.8	1.3	0.8
Paraplegia		0.2		<0.1
Lung circulation disorders	0.3	-	0.2	-
Dementia	0.3	-	0.2	-
Other neuro diseases except Parkinson’s	1.2	-	1.0	-
Obesity	6.9	-	2.5	-
Fluid and electrolyte disorders	1.1	1.1	0.4	<0.1
Alcohol abuse	2.0	-	0.7	-
Drug abuse	0.1	-	0.1	-
Depression	2.8	-	1.0	-
Parkinson’s disease	0.4	0.6	0.3	0.1
Diabetes	-	12.9	-	0.4
Rheumatic disorders	3.7	-	0.6	-
Polyarthrosis	5.3	4,3	0.8	-
Rheumatoid arthritis	0.5	0.9	0.2	-
Trauma	0.2	n/a	0.2	n/a
Other	7.2	4.7	4.5	6.2
Coxarthrosis / gonarthrosis	86.7	90.2	n/a (reference)	n/a (reference)

“-”indicates that the variable was not statistically significant at 5% for that joint replacement procedure and hence the PAF was not calculated

PFR = patellofemoral replacement. UKR = unicondylar knee replacement

### Hip replacement

Patients aged under 60 were the most likely to need an early revision, though PAFs were low. Males were 19% more likely than females to need earlier revision. The PAF for males was larger than for age at 7.2%.

Resurfacing was nearly twice as likely to require early revision than cemented (OR 1.82). Cementless HR had a 34% greater odds. Partial HR had higher odds, though a partial cemented replacement was not statistically different from a total cemented. Resurfacing had a PAF of 1.6% and uncemented HR had a PAF of 13.8%.

Other predictors of early revision were primary diagnosis of the index admission, previous all-cause emergency admissions and twelve comorbidities–all with PAFs of at most 2.5%. For the primary diagnosis of the index admission, significantly higher odds were seen for polyarthrosis, rheumatoid arthritis, trauma (fractured neck of femur) and other, the latter largely explained by osteonecrosis and non-union of fracture (pseudoarthrosis). Socio-economic status was not significant at p<0.05.

### Knee replacement

Patients aged under 60 were most likely to need an early revision. Those aged 40 to 44 years had the highest odds ratio at 1.63, but the PAF was highest for those aged 55 to 59 (1.2%). Males were 24% more likely than women to need early revision (PAF 7.1%).

Patellofemoral joint replacement conferred a significantly greater risk of early revision (OR 5.16, and the largest PAF of 7.6%), as did a partial knee replacement (OR 2.81, PAF 9.4%).

Other predictors of early revision were primary diagnosis of the index admission, previous all-cause emergency admissions, socioeconomic status and six comorbidities, with a lower odds for renal disease (OR 0.82). For the primary diagnosis of the index admission, significantly higher odds were seen for the “other” group only (compared with gonarthrosis). This was largely explained by the ICD10 code M2556 (pain in knee, with revision rate 2.5%) and several T84 codes (complications of internal orthopaedic prostheses, implants and grafts, mostly “other” and “mechanical” complications, with revision rates of around 20%). PAFs for these predictors were small.

## Discussion

The most important predictors of early revision (OR>2) were multiple previous emergency admissions (both joints), drug abuse (HR), and patellofemoral or partial joint replacement (KR). Age was also important, with younger people having the highest risk of revision and the elderly having the lowest, even after accounting for mortality. The primary diagnosis during the index admission was also a significant predictor. For hips, this was due to higher odds of revision for polyarthrosis, rheumatoid arthritis, fractured neck of femur and osteonecrosis; for knee, this was explained mostly by complications of previous implants. At a population level the most important factors (PAF>5%) where there was a substantial prevention potential were uncemented total hip replacements, and partial knee or patellofemoral replacements. Deprivation and comorbidity effects were modest.

### Comparison with other studies

Younger age in our study was associated with a greater risk for early revision in both hip and knee replacement surgery, consistent with other studies [26–30]. This has partly been thought to be due to polyethylene wear causing loosening in younger, more active patients, although this usually results in late wear [8,31]. On the other hand, confounding variables such as co-existing diagnoses may explain the inclusion of this factor. Age should be a factor included in the conversation when patients are counselled about the risk of early revision in primary arthroplasty. At a population level, the PAFs indicate that this is of modest importance.

Like ours, prior studies have identified males as carrying a higher risk of early revision [14,26,27]. This has been attributed to a higher rate of dislocations in males than females for HR [27]. There is no such apparent explanation with regards to early revision in males undergoing KR. One study has contextualised it with KR being increasingly performed in younger males [14]. It is recommended that this risk be explained in the consent process. From a population perspective, sex has an important impact, second only to procedure type.

Regarding the procedure subtype, Australian and New Zealand databases have similarly shown significantly higher rates of revision in partial KR [32,33]. On the other hand, a study using NJR data found smaller differences between the revision rates of partial KR versus Total Knee Replacement [34]. The authors believed this to be due primarily to progression of the degenerative process. Other reasons could include poor patient selection for partial KR and the threshold for revision for partial KR being lower than that for total KR as it is easier to revise.

In HR, we found that resurfacing and uncemented HR had higher revision rates than cemented HR, similar to a Swedish study, which found poorer performance of uncemented cups and unrecognised intraoperative femoral fractures [28]. Kandala et al was a UK-based study using NJR data that also confirms that cementless hip replacements needed earlier revision than cemented prostheses [35]. There is a large variation in practice, particularly in Nordic registries, with likely reasons including a lack of randomised controlled trial evidence, lenient regulatory requirements and intensive marketing [35]. Furthermore, Conroy et al, who investigated the Australian Joint Registry for those factors associated with revision for dislocation following primary HR, showed the same results as our study. They demonstrated a relative risk of 2 for fractured neck of femur and rheumatoid arthritis and 1.5 for avascular necrosis [36].

From the population perspective based on our data, the PAF was 13.8% for the uncemented prosthesis and 1.6% for resurfacing. This means that preventative measures at the population level should be targeted at uncemented arthroplasty. On the other hand, individual surgeons may have satisfactory outcomes with the type of HR to which they have become accustomed. Therefore, a change in practice might lead to worse outcomes.

Our study found higher rates of early revision in KR in areas with the lowest socioeconomic status at p<0.05. Dy et al also found this to be the case for KR but not HR [13,14]. Their finding suggested the need for higher levels of social support in impoverished areas to improve post-operative outcomes. However, others have demonstrated increased rates of revision in higher education levels [37]. This was thought to be due to more post-operative visits amongst this group for discussion of their progress and hence potentially earlier identification of patients who would benefit from revision [38,39].

Patient co-morbidities are perhaps the most relevant for the surgeon to consider when offering primary arthroplasty. Of our comorbidities that were highly significantly (p<0.01) associated with early revision, only Parkinson’s disease was common to both HR and KR. The higher number of significant comorbidities in HR could be due to the higher risk of dislocation in cases of neurological disorders, alcohol and drug abuse, hence the need for early revision. The outcome rates for the two joints were similar and the number of procedures for KR was higher than for HR, which argues against statistical artefact as an explanation for this difference. Depression has also been linked to early revision in other studies, thought to be due to lower levels of satisfaction and pain relief postoperatively[40]: we found as association for HR but not for KR regarding depression.

It is interesting that diabetes was not found to be relevant in HR, but significant in KR, while the converse was true for obesity. It is possible that the differences do exist but we were unable to detect them due to how these factors were recorded. Obesity in our database was dichotomous, i.e. either recorded or not, and dependent on ICD10 coding. The BMI was not recorded, which would have offered greater statistical power to detect associations. Diabetes was dichotomous for the same reason, with HbA1c lacking in the database, but the type and level of glycaemic control can determine the risk of infection and need for early revision [13,14]. Another research group also found obesity to be only linked to an increased risk in the HR population and explained this difference in a similar fashion [15,16]. The causal mechanism is thought to be the greater biomechanical load exerted in obese patients leading to early failure and an increased risk of infection in poorly controlled diabetic patients [14,41].

From a population perspective, we found that comorbidities are less important than the type of operation. The most relevant were obesity in HR (PAF 2.5%) and chronic lung disease in KR (0.8%). Although this is of modest importance at the population level, an awareness of these comorbidities is crucial, particularly in the pre-assessment clinic and the consent process when explaining to the patient their risk of early revision. It enables better patient selection as both the patient and the surgeon are informed of the relevant factors and the degree to which they are important in the need for early revision. It also allows targeted pre-operative optimisation of patients with these particular disorders.

Finally, in both HR and KR, early revision was likelier with increasing numbers of pre-operative emergency admissions. These could relate to the hip or knee pain, an unrelated new issue or a known comorbidity. This factor has to our knowledge not been identified in previous studies. It could reflect a combination of the previously mentioned factors, as patients with comorbidities are more likely to need to present to the emergency department. It could relate to various other patient characteristics, e.g. lower satisfaction levels [37,40]. Omitting this variable from the models had little impact on the size of the odds ratios, indicating an effect independent of our set of comorbidities.

### Limitations

Our study carries limitations inherent to the use of national administrative data. The accuracy of the primary diagnosis and procedure fields in HES is very high at 96% [42], and there are financial incentives to capture every admission. Secondary diagnosis coding is known to be less complete and vary by comorbidity, though studies of the relation between comorbidity and outcomes have shown that this underrecording does not lead to bias in the odds ratios [43]. We have already mentioned the lack of BMI and HbA1c. Joint laterality was 99% complete, but with administrative data, it is difficult to reflect case complexity. We had to exclude a small proportion of records due to invalid consultant codes but have no reason to think that the relations between predictors and early revision would differ greatly between those included and those excluded.

Unlike some administrative data sets in North America, for instance, HES data lack present-on-admission (POA) flags, i.e. they cannot distinguish between comorbidities and complications. While we tried to minimise this issue by using dementia, Parkinson’s disease plus the Elixhauser set (devised specifically for databases with no POA information), a minority of our flags such as renal disease and fluid and electrolyte disturbances could have occurred after the index procedure for some patients. This would limit their usefulness in pre-operative management.

We could not assess factors such as operative times and hospital volume, although it could be argued that in the case of the latter there is a more uniform distribution due to a nationalised health service in the UK [14,44]. Early revision is not always a true indicator of failure of primary procedure as patients may not seek advice or be offered revision. They may decline from other comorbidities and be unable to undergo the procedure, which is not reflected in our study. Analysis of the failure rate at specific post-operative intervals would be helpful, as the cause of failure within 30 days may be very different from that at 3 years. Furthermore, our data do not describe the extent to which each clinician excluded extra-articular causes of postoperative pain such as vascular pathologies, bursitis, iliotibial band syndrome and psychological illness, which have previously been described in patients following primary arthroplasty [45].

Finally, as there is evidence that revision due to dislocation or infection may have different risk factors [46] our analysis could be extended to examine the risk factors for different primary diagnoses for the revision admission separately.

## Conclusions

Meeting the rising demand for revision surgery in the UK is a challenge for leaders in healthcare and policymakers. Our study is the first to use UK national administrative data to identify risk factors for early revision surgery. From a population perspective, the effects of socio-economic deprivation and comorbidity are dwarfed by those of procedure subtype. We recommend developing strategies to optimise patients pre-operatively and to improve patient selection for primary arthroplasty with a better informed consent process.

## Supporting information

S1 Table OPCS procedure codes from the National Joint Registry: “NJR report hip and knee OPCS codes.” (DOCX)
