# Supplementary material for: Risk factors for early revision after total hip and knee arthroplasty: National observational study from a surgeon and population perspective
Source: PLoS One. 2019 Apr 9;14(4):e0214855. doi: 10.1371/journal.pone.0214855 (PMC6456180; doi:10.1371/journal.pone.0214855)
Supplement: S1 Table — (DOCX) [file pone.0214855.s001.docx]

S1 Table. Procedure codes for hip and knee replacement

NJR report hip and knee codes

<http://www.njrcentre.org.uk/njrcentre/Portals/0/Documents/OPCS4%20Procedure%20Codes%20used%20in%20NJR%20Annual%20Report.pdf>

Hips

Codes:

W371

W378

W379

W381

W388

W389

W391

W398

W399

W521 And (Z843 Or Z761 Or Z756)

W531 And (Z843 Or Z761 Or Z756)

W541 And (Z843 Or Z761 Or Z756)

W581 And (Z843 Or Z761 Or Z756)

W931

W938

W939

W941

W948

W949

W951

W958

W959

Descriptions:

W371 Primary total prosthetic replacement of hip joint using cement

W378 Total prosthetic replacement of hip joint using cement, Other specified

W379 Total prosthetic replacement of hip joint using cement, Unspecified

W381 Primary total prosthetic replacement of hip joint not using cement

W388 Total prosthetic replacement of hip joint not using cement, Other specified

W389 Total prosthetic replacement of hip joint not using cement, Unspecified

W391 Primary total prosthetic replacement of hip joint NEC

W398 Other specified hybrid prosthetic replacement of hip joint using cemented acetabular component

W399 Unspecified hybrid prosthetic replacement of hip joint using cemented acetabular component

W521 Primary prosthetic replacement of articulation of bone using cement NEC

W531 Primary prosthetic replacement of articulation of bone not using cement NEC

W541 Primary prosthetic replacement of articulation of bone NEC

W581 Primary resurfacing arthroplasty of joint

W931 Primary hybrid prosthetic replacement of hip joint using cemented acetabular

component

W938 Other specified hybrid prosthetic replacement of hip joint using

cemented acetabular component

W939 Unspecified hybrid prosthetic replacement of hip joint using cemented

acetabular component

W941 Primary hybrid prosthetic replacement of hip joint using cemented femoral

component

W948 Other specified hybrid prosthetic replacement of hip joint using cemented femoral component

W949 Unspecified hybrid prosthetic replacement of hip joint using cemented femoral component

W951 Primary hybrid prosthetic replacement of hip joint using cement NEC

W958 Other specified hybrid prosthetic replacement of hip joint using cement

W959 Unspecified hybrid prosthetic replacement of hip joint using cement

Auxiliary codes mentioned above:

Z843 Hip joint

Z761 Head of femur

Z756 Acetabulum

Knees

Codes:

O181

O188

O189

W401

W408

W409

W411

W418

W419

W421

W428

W429

W521 And (Z846 Or Z765 Or Z845 or Z844 Or Z774 Or Z787)

W531 And (Z846 Or Z765 Or Z845 or Z844 Or Z774 Or Z787)

W541 And (Z846 Or Z765 Or Z845 or Z844 Or Z774 Or Z787)

W581 And (Z846 Or Z765 Or Z845 or Z844 Or Z774 Or Z787)

Descriptions:

O181 Primary Hybrid Prosthetic Repl Knee Joint Using Cement

O188 Hybrid Prosthetic Replacement Knee Joint Using Cement, Other Specified

O189 Hybrid Prosthetic Replacement Knee Joint Using Cement, unspecified

W401 Primary total prosthetic replacement of knee joint using cement

W408 Total prosthetic replacement of knee joint using cement, Other specified

W409 Total prosthetic replacement of knee joint using cement, Unspecified

W411 Primary total prosthetic replacement of knee joint not using cement

W418 Total prosthetic replacement of knee joint not using cement, Other specified

W419 Total prosthetic replacement of knee joint not using cement, Unspecified

W421 Primary total prosthetic replacement of knee joint NEC

W428 Other total prosthetic replacement of knee joint, Other specified

W429 Other total prosthetic replacement of knee joint, Unspecified

W521 Primary prosthetic replacement of articulation of bone using cement NEC

W531 Primary prosthetic replacement of articulation of bone not using cement NEC

W541 Primary prosthetic replacement of articulation of bone NEC

W581 Primary resurfacing arthroplasty of joint

Auxiliary codes used above:

Z846 Knee joint

Z765 Lower end of femur NEC

Includes: Articular surface of femur at knee

Z845 Tibiofemoral joint

Z844 Patellofemoral joint

Z774 Upper end of tibia NEC

Includes: Articular surface of tibia at knee

Z787 Patella
